# Selected Formulæ

**Published:** 1892-06

**Authors:** 


### SELECTED FORMULÆ.

*Treatment of Pruritus Vulvæ.*—Dr. J. B. Kessler, of Iowa City, writes: "I noticed in the *Medical Record* of April 30, 1892, treatment of pruritus vulvæ by galvanism. I have used for many years hydrarg. corrosiv. chloride for the same; also for pruritus ani. I formerly used it in alcoholic solution, but latterly in tincture of myrrh, gr. ij—iv to ℥j. After cleansing the parts thoroughly with hot water, apply with sponge or probe, wrapped with cotton; two or three applications is usually suffi-

*Centralblatt fur Chirurgie, 1891, p. 894.

†St. Petersburger Med. Wochenschrift, 1884.

cient. I might add that the tincture of myrrh is not original, but read of it several years ago from Prince A. Morrow, M. D., of New York, recommending it as a vehicle in treating tænia circinatus.—*N. Y. Med. Record.*

---

*Acne.*—The following is said to have been very serviceable in acne:

℞ Sulph. præcip .................... gr. xv
Spt. camphoræ .................... gtt. v
Glycerini .......................... ʒ ss
Aq. calcis .......................... ℥ j

Sig. Apply freely at night, and wash off in the morning.

A 1 to 1,000 solution of corrosive sublimate in emulsion of almond oil is also recommended in this condition.

---

*Locomotor Ataxia.*—A modification of the suspension treatment of tabes consists in the forcible flexion of the thighs of the abdomen, the knees being held extended. The maneuver sometimes causes severe pain, but no other untoward symptom has been reported from its use.—*Ex.*

---

*Treatment of Diphtheria in Paris.*—The following treatment of diphtheria in children is much advocated at present. The room is steamed with a solution as follows:

Phenic acid .......................... ℥ ij

Salicylic acid .......................... ʒiv

Benzoic acid .......................... ℥jss

Alcohol .......................... ℥iv

A tablespoonful is put into a quart of boiling water, and re-renewed every three hours.

As a local application:

Camphor .......................... ʒ v

Castor oil .......................... ʒ iv

Alcohol .......................... ʒ iij

Phenic acid .......................... ʒ j

Tartaric acid .......................... ℈ j

—*Medical Press.*

---

*Injection for Gleet.*—

℞ Hydrarg. bichlorid .............. gr. ½

Zinci sulph. carbolat................ ʒ ss

Acid boric.......................... ʒ jss

Liq. hydrogen peroxid ............ f ℥ iv

Aquæ destillat.................. ad f ℥ vj

M. Use injection in the morning and evening.

—*Dr. A. Hewson, College and Clinical Record.*

